# Factors Associated With Overweight and Obesity Among Mexican Americans and Central Americans: Results From the 2001 California Health Interview Survey

**Published:** 2006-12-15

**Authors:** Janice V Bowie, Hee-Soon Juon, Elisa M Rodriguez, Juhee Cho

**Affiliations:** Johns Hopkins University Bloomberg School of Public Health. Dr Bowie is also affiliated with the Center for Health Disparities Solutions.; Department of Health, Behavior and Society, Johns Hopkins University Bloomberg School of Public Health; Department of Health, Behavior and Society, Johns Hopkins University Bloomberg School of Public Health; Department of Health, Behavior and Society, Johns Hopkins University Bloomberg School of Public Health

## Abstract

**Introduction:**

Hispanics are the fastest growing demographic group in the United States; however, "Hispanic" is a broad term that describes people who are from or whose ancestors are from multiple countries of origin. This study examines, separately, the social, cultural, and behavioral factors associated with overweight and obesity among Mexican American adults and among Central American adults.

**Methods:**

To estimate the prevalence of overweight and obesity among Mexican and Central Americans living in California, we conducted a cross-sectional analysis of data from the 2001 California Health Interview Survey using SUDAAN software to account for the survey's multistage sampling design.

**Results:**

Of the 8304 Mexican Americans participating in the survey, 36.8% were overweight and 26.2% were obese. Of the 1019 Central Americans, 39.2% were overweight and 22.2% were obese. Among Mexican American men, age and marital status were associated with overweight and obesity; and education, acculturation, health insurance status, health status, and use of vitamins were associated with obesity only. Among Mexican American women, age, education, number of children, health status, and health behavior were associated with overweight and obesity. Among Central American men, age, education, and access to health care were associated with overweight, whereas marital status, acculturation, health care, and binge drinking were associated with obesity. Among Central American women, number of children was associated with overweight and obesity; and age and education were associated with obesity only.

**Conclusion:**

Our findings of high rates of overweight and obesity among Mexican and Central Americans in California indicate the need for a wide variety of effective weight-loss interventions targeting these populations, and the differences we found in the factors associated with overweight and obesity may suggest the need for unique intervention strategies for different Hispanic subgroups.

## Introduction

The prevalence of excess weight is increasing rapidly across the country: close to 65% of the U.S. adult population was recently estimated to be overweight or obese (1-2). According to data from the National Health and Nutrition Examination Survey (NHANES), the combined prevalence of overweight and obesity (body mass index [BMI] ≥25kg/m^2^) increased by nearly 40% between 1976–1980 and 1999–2000 (from 46% to 64.5%), and the prevalence of obesity (BMI ≥30kg/m^2^) increased by 110% (from 14.5% to 30.5%) (2).

Although the prevalence of obesity has increased among Americans of all ages, races, ethnicities, socioeconomic levels, and geographic areas (3-4), one of the largest increases has occurred among Hispanics: from 12% in 1991 to 21% in 1998 (5). Part of this increase in the prevalence of obesity among Hispanics may be attributable to changes in dietary habits, which have been well documented among Hispanics as a group, although few studies have been reported specifically on the dietary habits of U.S. Hispanics of Central American ancestry. The Hispanic Health and Nutrition Examination, for example, sampled only Mexican Americans, Cuban Americans, and Puerto Ricans, and NHANES III, conducted from 1989 to 1994, sampled only Mexican Americans (2).

Demographic indicators of low socioeconomic status (SES), including low educational attainment, unemployment, poverty, and (for women) number of children, have previously been associated with overweight and obesity among members of specific ethnic groups (6-9). Lack of health insurance coverage and restricted access to health care services, including services for the prevention, treatment, and management of conditions related to overweight and obesity, have also contributed to Hispanics' relatively poor health status. Approximately 32.4% of the 45 million uninsured people in the United States are Hispanic (10). Hispanics' efforts to acquire health insurance and health care have sometimes been compromised by language barriers and cultural practices and beliefs (11).

For immigrant populations, acculturation to U.S. norms can lead to the adoption of a more sedentary (westernized) lifestyle and, as a result, to excess weight and obesity (6,7,12,13). Acculturation occurs when members of one cultural group (usually a minority group) adopt the beliefs and behaviors of another group (usually the dominant group) (14). For the past 15 years, the prevalence of obesity among immigrants living in the United States has approached that of U.S.-born adults even though most minority immigrants in the United States are from countries where the prevalence of obesity is lower than in the United States (6).

Overweight and obesity are associated with significant health problems and financial burdens. In the United States, obesity was recently estimated to be responsible for almost 300,000 deaths each year and annual health care costs of $117 billion (1). In 2000, more than 17% of all deaths in the United States were attributable to overweight and obesity; only tobacco use accounted for more deaths (15). Overweight and obesity have also been linked to a variety of chronic diseases, including cardiovascular disease, type 2 diabetes, hypertension, stroke, dyslipidemia, osteoarthritis, breathing problems, some cancers, and depression (16).

Overweight and obesity are caused by an imbalance between the number of calories that people consume and the number of calories that they burn (17). According to the Centers for Disease Control and Prevention, poor diet and physical inactivity, both modifiable behaviors, are substantial contributors to deaths associated with being overweight (6). While factors such as genetics and aging may also increase people's risk of being overweight, in the United States the environment is believed to be influential in promoting energy intake and discouraging physical activity (17,18).

For this study, we analyzed data from the 2001 California Health Interview Survey (CHIS 2001) to examine how various social, cultural, and behavioral factors were related to overweight and obesity among the adult Hispanic population as a whole and the Mexican American and Central American populations specifically. The findings from this study can add to our understanding of how these factors may influence Mexican Americans' and Central Americans' response to obesity prevention interventions.

## Methods

### Data source

CHIS 2001 is a collaborative project of the UCLA Center for Health Policy Research, the California Department of Health Services, and the Public Health Institute. The largest health survey ever conducted in any state, it sampled 55,428 households randomly drawn from every county in California and was administered through random digit dialing (RDD). The survey was designed to produce reliable estimates of various health parameters for all counties in the state, including medium- and small-sized ones (19). It provided estimates for California's overall population, as well as for several of the state's racial/ethnic groups, and was administered in both English and Spanish.

For this study, we categorized Hispanic survey participants as being either Mexican American or Central American. Those in the Central American category consisted of respondents identified as Salvadorian, Guatemalan, Costa Rican, Honduran, Nicaraguan, Panamanian, Central American, or Belizean.

### Measures

We assessed the relationship between the prevalence of overweight and obesity and four categories of factors: demographic characteristics (age, marital status, number of children) (6,7); socioeconomic factors (education, employment status, poverty status) (6,7,12); acculturation (5,20); access to health care (insurance and general health status) (5,6,10,21); and health-related behavior (drinking alcohol, smoking, eating five or more servings of fruits and vegetables a day, taking vitamins) (5,9,21). We used respondents' BMI to determine their weight status. BMI is calculated by dividing a person's weight in kilograms by the square of that person's height in meters; a person with a BMI of 25–29.9 is considered overweight, and a person with a BMI of ≥30 is considered obese (22).

### Sociodemographic categories

We divided all respondents into five age categories (18–29, 300–39, 400–49, 50–64, and 65 or older) and three marital status categories (married, separated/divorced/widowed, and never married).  We also divided female respondents into five categories based on the number of children they had (0, 1, 2, 3, and 4 or more). 

For SES indicators, we used three educational attainment categories (college graduate, high school graduate/some college, and less than high school graduate), two employment categories (employed and unemployed), and four family income levels expressed as a percentage of the federal poverty level (FPL) (≥300%, 200-299%, 100-199%, and ≤99%). The FPL is adjusted to account for family size (21).

### Acculturation

As proxy measures of acculturation, we used duration of U.S. residence and spoken English proficiency in our original model. However, because duration of residence and spoken English proficiency were highly associated (χ^2^ = 5491.2, df = 12, p<.001), we included only duration of U.S. residence in the final model. In the bivariate analysis, we found no significant differences in BMI among respondents who had been in the United States less than 5 years, those who had been there 5 to 9 years, and those who had been there 10 to 14 years. Therefore, we divided respondents into only two duration of U.S. residence categories (more than 15 years or U.S. born, and less than 15 years).  

### Access to health care and health status

We used respondents' health insurance status (did or did not have) to assess their access to health care. We also asked them to rate their general health status on a 5-point scale (from 5 [poor] to 1 [excellent]), and we used this rating as a continuous variable in our analyses.

### Health behaviors

We divided respondents into two categories by their current smoking status, binge-drinking status, whether they ate at least 5 servings of fruits and vegetables a day, and whether they took vitamins. We divided them into three categories based on the number of times per week that they reported engaging in vigorous activity (3 or more, 1 or 2, and none). Respondents who smoked when the survey was administered were categorized as current smokers, and those who had either quit smoking or never smoked regularly were categorized as nonsmokers. Binge drinking was defined as having five or more drinks on at least one occasion during the previous month. Vigorous activity was defined as engaging in leisure-time activity that caused heavy sweating or a large increase in breathing or heart rate for at least 10 minutes during the preceding 30 days.

### Data analysis

We first conducted bivariate analyses to determine which independent variables were associated with overweight and obesity. We then tested interaction terms between variables on the basis of previous study results and behavioral plausibility. Finally, we used multivariate logistic regression (MLR) analyses to identify salient predictors of overweight and obesity while controlling for variables in sociodemographic characteristics, acculturation, access to care, and health behavior.

We weighted the descriptive and logistic regression analyses by using SUDAAN (Survey Data Analysis, Research Triangle Institute, Research Park Triangle, NC) to account for the design of the complex, multistage sample, and we used the jackknife method to compute standard error estimates (23). We performed separate analyses by sex as well as by Hispanic subgroup.

## Results

### Characteristics of Hispanics in California

Table 1 presents information about Mexican Americans and Central Americans participating in the 2001 CHIS. The 9460 eligible respondents represented an estimated 6,101,852 Hispanic adults residing in California. Mexican Americans (87.8%) and Central Americans (10.8%) were the largest Hispanic subgroups in the CHIS.

Among Mexican American respondents, the mean age was 36.7 years, the age range was 18 to 105 years, and about 51% were male. Overall, Mexican Americans had low levels of educational attainment, and two thirds were currently employed. More than half were married, and nearly a quarter had never been married. More than a third reported incomes at or below the poverty level. One third reported being in fair to poor health condition, and two thirds had health insurance. About half were not U.S. citizens, and about 27% were U.S. born. More than half reported difficulty speaking English.

Among Central American respondents, the mean age was 37.6 years, the age range was 18 to 88 years, and almost half were male. About 60% of Central Americans had less than a high school education, and two thirds were employed. Almost 50% were married, and 24% had never been married. Only 15% reported incomes at or below the poverty level, and almost 44% reported incomes at or above 300% of the poverty level. Only 7% were born in the United States, and almost two thirds were not U.S. citizens. About two thirds reported difficulty speaking English. We found no statistically significant differences between Mexican and Central Americans with respect to age, education, marital status, or employment status; however, Central Americans were significantly less likely to have incomes below the poverty level or to be U.S. citizens.

### Prevalence of overweight and obesity by sex

Approximately 36.8% of Mexican Americans were overweight, 24.2% were obese, and 8.6% did not report their body weight or height. As shown in Table 2, Mexican American men were much more likely to be overweight than Mexican American women (44.2% vs 29.0%), though women were slightly more likely to be obese (25.1% vs 23.3% ) and also more likely not to report their body weight or height (11.9% vs 5.4%).

About 39.4% of Central Americans were overweight, 22.2% were obese, and the BMI for 7.5% could not be calculated because of missing height or weight data. As with Mexican Americans, men were more likely to be overweight (50.6% vs 28.4%), and women were more likely to be obese (26.5% vs 17.8%) and not to report their weight or height (11.7% vs 3.2%).

### Prevalence of overweight and obesity by age

As shown in Table 3, the weight status of both Mexican and Central Americans generally increased with age through age 40 to 49. Among Mexican Americans, those in their 40s were most likely to be overweight, followed by those in their 30s; and those aged 50 to 64 were most likely to be obese, followed by those in their 40s. Among Central Americans, those in their 30s were most likely to be overweight, followed by those in their 40s; and those in their 40s were most likely to be obese, followed by those aged 50 to 64.

### Prevalence of overweight and obesity by social, behavioral, and cultural factors

**Mexican American men**

As shown in Table 4, both age and marital status were associated with being overweight among Mexican American men. As mentioned previously, those aged 30 to 39 (OR, 2.12; 95% CI, 1.54–2.92), 40 to 49 (OR, 2.35; 95% CI, 1.58–3.48), and 50 to 64 (OR = 2.52; 95% CI, 1.57–4.05) were more likely to be overweight than those aged 18 to 29. Single men were less likely than married men to be overweight (OR, 0.71; 95% CI, 0.52–0.97).

Age and marital status were also associated with obesity among Mexican American men. Those aged 30 to 39 (OR, 1.97; 95% CI, 1.44–2.70), 40 to 49 (OR, 2.58; 95% CI, 1.88, 3.53), and 50 to 64 (OR, 3.69; 95% CI, 2.56–5.31) were more likely to be obese than those aged 18 to 29, and single men were less likely to be obese than married men (OR, 0.68; 95% CI, 0.46–0.99). Obesity was also associated with education attainment, access to health care, acculturation level, self-reported health status, and use of vitamin supplements among Mexican American men. Those who had not completed high school were more likely to be obese than were those who had graduated from college (OR, 2.34; 95% CI, 1.39–3.94); those who were born in the United States or had lived there for more than 15 years were more likely to be obese than those who had lived there for less than 5 years (OR, 1.36; 95% CI, 1.01–1.87); those with health insurance were more likely to be obese than those without; those who reported being in poor health were more likely to be obese than those who did not (OR, 1.66; 95% CI, 1.44–1.91); and those who reported taking no vitamins were more likely to be obese than those who reported taking them (OR, 1.31; 95% CI, 1.07–1.62).

**Mexican American women**

Among Mexican American women, age, education, number of children, health status, and lack of involvement in weekly vigorous activity were associated with being overweight. Those aged 30 to 39 (OR, 1.49; 95% CI, 1.10–2.03), 40 to 49 (OR, 1.63; 95% CI, 1.19–2.23), and 50 to 64 years (OR, 2.00; 95% CI, 1.35–2.98) were more likely to be overweight than those aged 18 to 29; those who had not finished high school were more likely to be overweight than college graduates (OR, 1.44; 95% CI, 1.02–2.04) or those who had completed high school (OR, 1.61; 95% CI, 1.09–2.39); those with three children (OR, 1.81, 95% CI, 1.18–2.78) and those with more than four children (OR, 1.78, 95% CI, 1.11–2.85) were more likely to be to be overweight than those with no children. Being in poor general health (OR, 1.18; 95% CI, 1.05–1.34) and not engaging in vigorous physical activity (OR, 1.51; 95% CI, 1.15–1.98) were also associated with being overweight. 

Obesity among Mexican American women was associated with age, education, number of children, access to health care, general health condition, and health behavior. Those in their 30s, 40s, and aged 50 to 64 all had a higher risk of being obese than those aged 18 to 29; those with less than a high school education were more likely to be obese than college graduates (OR, 1.96; 95% CI, 1.14–3.37); those with four or more children were more likely to be obese than those who had no children (OR, 1.91; 95% CI, 1.20–3.04); those with health insurance were more likely to be obese than those without health insurance; those who reported poor health were more likely to be obese than those who did not (OR, 1.64; 95% CI, 1.43–1.89); and those who reported taking no vitamins were more likely to be obese than those who reported taking them (OR, 1.28; 95% CI, 1.04–1.55).

**Central American men**

Among Central American men, age, education, and access to health care were associated with being overweight (Table 5). Those aged 30 to 39 (OR, 2.71; 95% CI, 1.25–5.88) and those over age 65 (OR, 10.41; 95% CI, 2.16–50.14) were more likely to be overweight than those aged 18 to 29. Interestingly, those with less than a high school education were less likely to be overweight than those with more than a college education (OR, 0.32; 95% CI, 0.10–0.99), and those with no health insurance were more likely to be overweight than those with health insurance (OR, 2.21; 95% CI, 1.15–4.24): both of these associations with being overweight were the reverse of what we found among Mexican American men.

Marital status, acculturation, access to health care, and binge drinking were associated with obesity among Central American men. Those who were single were less likely to be obese than those who were married (OR, 0.17; 95% CI, 0.05–0.58); those who were born in the United States or had lived here for more than 15 years were about 5 times more likely to be obese (OR, 4.92; 95% CI, 1.64–14.74) than those who had lived here for less than 15 years; those who had no health insurance were more likely to be obese than those with health insurance (OR, 3.80; 95% CI, 1.45–9.95); and those who reported binge drinking were less likely to be obese than those who did not (OR, 0.43; 95% CI, 0.18–0.99).

**Central American women**

Among Central American women, number of children was the only factor associated with being overweight: those with three children were at significantly greater risk of being overweight than those with no children (OR, 2.80; 95% CI, 1.11–7.04). However, age, education, and number of children were all associated with obesity among Central American women. As might be expected, those in their 40s (OR, 7.76, 95% CI, 3.57–16.87) and those aged 50 to 64 (OR, 3.06; 95% CI, 1.08–8.64) were more likely to be obese than those aged 18 to 29. Somewhat surprisingly, however, Central American women who were high school graduates were less likely to be obese than those who were college graduates (OR, 0.30; 95% CI, 0.11–0.80), and those with two children were less likely to be obese than those with none (OR, 0.32; 95% CI, 0.11–0.94).

## Discussion

Our findings substantiate those of previous studies showing that Mexican American adults living in the United States have relatively high rates of overweight and obesity. For example, 1999–2002 data from NHANES showed that 73% of Mexican American adults were at least overweight and 33% were obese; they also showed that obesity rates had increased from 24% to 27% among Mexican American men and from 35% to 38% among Mexican American women during this period (24). Our findings, however, also include some disaggregated information on Central Americans, who do not routinely appear in the health literature, and highlight clear differences between Mexican Americans and Central Americans in how various factors affect their risk of being overweight or obese.

In all four ethnic subgroup/sex categories, increasing age was associated with an increased risk of being overweight or obese, at least through age 49. Education level was inversely associated with the risk of being overweight or obese for all Mexican American groups, though the high rate of obesity among Mexican Americans who did not complete high school was particularly compelling. Among Central Americans, those who were college graduates were somewhat surprisingly more likely to be overweight or obese than were those who did not have a college education. Using data from NHANES III, Zhang and Wang (25) found that overweight was more prevalent among women than among men; by age, most prevalent among adults aged 41–49; and among men more prevalent among those of high SES but among women more prevalent among those of low SES. Results from the San Antonio Heart Study (26)  showed that the prevalence of overweight was higher among Hispanics than among non-Hispanic whites and higher among women than men.

In our study, both Mexican American and Central American women with three children were more likely to be overweight than were those with none, while having four or more children was associated with obesity only among Mexican American women. The results of previous studies have similarly suggested a positive association between the number of children that women have and their risk of being overweight or obese (27-29).

We found that acculturation as indicated by U.S. residency of 15 years or more was a strong correlate of obesity for both Mexican and Central American men but not for women of either group. We also found that Mexican Americans, on average, had been in the United States longer than Central Americans. Previous research has indicated that level of acculturation may play an important role in the development of obesity within the Hispanic population, as immigrants follow the trend of native-born Americans toward more sedentary behavior and the consumption of more calorie-rich foods (30-32). Data from a cross-sectional survey of Latino men and women from a community sample indicated that acculturation was the strongest correlate of obesity (5), although other research findings also suggest that acculturation may be more complex than the definition we used and needs to be defined more broadly (33). We recommend that future studies of obesity risk and overall health in immigrant populations focus on how both are affected by the interplay of sociocultural and income changes (34).

We found that fair to poor self-reported health status was consistently associated with an increased risk of being overweight or obese. However, the correlation between access to care, as measured by having health insurance, and the risk of being overweight or obese varied substantially between Central and Mexican Americans. Mexican American men and women with health insurance were each more likely to be obese than those without, whereas Central American men without coverage (but not Central American women without coverage) were more likely to be overweight and more likely to be obese than were those with coverage. Further research will be needed to delineate the relationship between having health insurance and the risk of being overweight or obese in these two Hispanic subgroups.

Hispanics in general have self-reported comparatively poor access to health care services, which is usually associated with a lack of health insurance (11,34). Although the results of two studies have suggested that insurance status has no real effect on the quality of health care (30,35), the National Health Disparities Report, which offers a snapshot of the nation's progress on health care, showed that various health parameters, including insurance as a measure of access, were worse among Hispanics than among most other U.S. racial/ethnic groups (31). Furthermore, some of this disparity has been attributed to Hispanic men having particularly low rates of health insurance and health care access, which two recent studies have cited as being important factors in health outcomes (11,36). The findings from these studies suggest that although having health insurance may not ensure that people receive quality care, it at least makes accessing care easier. In addition, the health status and access to care of undocumented immigrant Hispanics are likely to be worse than those we reported among Hispanics who are here legally (11).

In analyzing health behaviors, we found that Central American men who reported binge drinking were less likely to be obese than those who did not. We also found that Mexican American men and women who did not take vitamins were more likely to be obese than those who did. Micronutrient deficiencies and poor dietary variety have been associated with high energy intake and BMI; and serum concentrations of certain vitamins such as A, D, E, and the carotenoids have been associated with diet, race, and obesity (37,38). Our findings thus support those of previous studies and suggest that greater emphasis should be placed on the role of diet and micronutrients in maintaining an optimal BMI, particularly among people such as binge drinkers, who are particularly vulnerable to dietary shortfalls.

Finally, we found a significant association between absence of physical activity and being overweight among Mexican American women. A substantial body of data has shown physical activity levels to be associated with body weight and body fatness (18,39). Two studies (40,41) have shown levels of physical activity to be lower among Hispanics than among non-Hispanic whites, and this lack of physical activity is a possible factor in the high rates of type 2 diabetes found among Hispanics, especially Mexican Americans (42). To increase levels of physical activity and exercise among particular Hispanic subgroups, health officials will need to examine barriers to and facilitators of such activity for each subgroup before planning strategies to enhance participation. And in planning strategies for women, they should also consider issues pertaining to body image and weight-related distress.

Overall, most of the factors we found associated with overweight or obesity have been reported in other studies; however, our study was notable in that it sampled a large number of Hispanics from California, where the majority of Mexican Americans and a substantial portion Central Americans in the United States reside. The large number of Mexican Americans in our study sample allowed us to estimate the prevalence of overweight and obesity and to identify the risk factors for excess weight in this population with considerable confidence, and the inclusion of Central Americans allowed us to present corresponding estimates for a segment of the U.S. Hispanic population that has been ignored in many studies and national surveys.

Because our analyses were based on cross-sectional data, significant associations with overweight or obesity should not be taken as proof of causation. Other limitations include incomplete dietary data on respondents' portion sizes and consumption of high-calorie foods such as sweets. Our study also did not address environmental factors that contribute to weight gain, such as a reliance on fast food outlets and convenience stores with limited dietary choices, and heavy marketing of calorie-dense foods (2,43,44). In addition, we calculated subjects' BMI on the basis of their self-reported height and weight, which may have led to the misclassification of some overweight or obese participants, since people who are overweight or obese may tend to underestimate their weight and overestimate their height (45). Although we used the normal BMI criteria for classifying people as overweight or obese, BMI calculations based on actual height and weight measurements rather than the self-reports of survey participants may have produced different results; however, the percentages of Mexican Americans aged 18 to 64 that we estimated to be obese using CHIS data were similar to corresponding estimates based on data from NHANES III, in which subjects' BMI was derived from clinical measurements of their height and weight (23% vs 20% for men; 25% vs 24% for women).

Because Hispanics are expected to constitute 25% of the U.S. population by 2050 (46), policymakers, community leaders and members, and Hispanic advocates must act now to reduce the striking health disparities between U.S. Hispanics and the overall U.S. population. Our findings show that a considerable proportion of Mexican Americans and Central Americans are at risk for adverse health outcomes because of their weight; however, they also show that rates of overweight and obesity in these two Hispanic subgroups vary, as do some of the factors associated with being overweight or obese. These differences suggest the need for weight-reduction interventions that target specific subgroups of Hispanics by country of origin, level of acculturation, and socioeconomic status; they also support previous recommendations that providers of health care information to Spanish-speaking populations consider factors such as their audience's cultural competency and language proficiency (43,47,48). Our finding that the percentage of Hispanics who are overweight or obese increases with acculturation also suggests that one approach to reducing rates of overweight and obesity and improving the overall health of Hispanics might be to encourage them to maintain their traditional dietary practices. However, whatever specific interventions are used to reduce the prevalence of overweight and obesity in Hispanic communities, the success of these intervention efforts will continue to depend on cooperation between community members and health care workers (5).

## Figures and Tables

**Table 1:** Selected Characteristics of Hispanic Adults Aged ≥18 Years, by Hispanic Subgroup, 2001 California Health Interview Survey

Characteristics	Mexican Americans (n = 8304) % (SE^a^)	Central Americans (n = 1019) % (SE^a^)	Total (n = 9460) % (SE^a^)
**Sex**			
Female	48.50 (0.47)	51.18 (1.99)	49.01 (0.27)
Male	51.50 (0.47)	48.82 (1.99)	50.99 (0.27)
**Education**			
<High school graduate	57.23 (0.57)	59.01 (2.22)	53.63 (0.48)
High school graduate	23.30 (0.57)	20.07 (1.74)	23.75 (0.45)
Some college	14.19 (0.41)	15.06 (1.50)	15.83 (0.36)
College graduate	5.28 (0.30)	5.86 (0.88)	6.78 (0.31)
**Employment status**			
Employed	65.96 (0.72)	67.78 (2.38)	66.11 (0.57)
Unemployed	34.04 (0.72)	32.22 (2.38)	33.89 (0.57)
**Marital status**			
Married	55.47 (0.76)	50.01 (2.07)	53.74 (0.67)
Separated/divorced/widowed	22.01 (0.75)	26.18 (1.72)	22.23 (0.68)
Never married	22.52 (0.58)	23.72 (1.65)	23.82 (0.49)
**Income as % of federal poverty level**			
<100%	36.70 (0.78)	14.91 (5.94)	34.96 (0.69)
100-199%	32.93 (0.70)	29.18 (6.02)	32.43 (0.67)
200-299%	14.17 (0.56)	11.72 (3.81)	13.85 (0.54)
>300%	16.20 (0.54)	44.19 (6.10)	18.76 (0.45)
**Self-reported general health status**			
Excellent	11.13 (0.47)	13.36 (1.71)	11.91 (0.44)
Very good	16.97 (0.63)	14.64 (1.29)	18.30 (0.56)
Good	38.83 (0.70)	36.05 (1.90)	37.82 (0.64)
Fair	28.77 (0.78)	30.76 (2.02)	27.53 (0.70)
Poor	4.29 (0.32)	5.20 (0.92)	4.44 (0.28)
**Health insurance status**			
Has health insurance	64.75 (0.79)	59.19 (2.14)	65.81 (0.65)
Does not have health insurance	35.25 (0.79)	40.85 (2.14)	34.19 (0.65)
**Citizenship**			
U.S. born	27.31 (0.58)	7.34 (0.97)	29.50 (0.54)
Naturalized	19.63 (0.51)	27.61 (1.74)	20.55 (0.51)
Not U.S. citizen	53.06 (0.73)	65.05 (1.98)	49.95 (0.65)
**Duration of U.S. residence**			
<5 years	8.33 (0.49)	9.91 (1.53)	7.89 (0.43)
5-9 years	11.54 (0.58)	10.99 (1.48)	10.52 (0.52)
10-14 years	19.34 (0.67)	25.10 (1.96)	18.22 (0.57)
≥15 years	33.38 (0.67)	46.61 (1.87)	33.75 (0.61)
U.S. born	27.42 (0.58)	7.40 (0.98)	29.62 (0.52)
**Spoken English proficiency**			
Not good	55.14 (0.73)	60.32 (2.06)	50.89 (0.63)
Good	18.05 (0.63)	22.57 (1.79)	19.05 (0.49)
Very good	17.03 (0.49)	14.34 (1.38)	19.43 (0.49)
English is native language	9.78 (0.40)	2.78 (0.75)	10.64 (0.38)

a SEs (standard errors) adjusted for design effect with SUDAAN.

**Table 2:** Prevalence of Normal Weight, Overweight, and Obesity Among Hispanic Adults, by Sex and Ethnic Subgroup, 2001 California Health Interview Survey

Body Mass Index (BMI) Classification	Men** % (SE^a^)**	Women** % (SE^a^)**	Total** % (SE^a^)**	χ^2^_3_ (*P*)
**Mexican American**				
Normal (BMI <25)	27.10 (1.00)	33.94 (0.99)	30.43 (0.67)	179.1 (<.001)
Overweight (BMI 25-29.9)	44.17 (0.97)	29.03 (0.77)	36.78 (0.63)	
Obese (BMI ≥30)	23.30 (0.97)	25.10 (0.84)	24.18 (0.68)	
Data missing	5.43 (0.52)	11.94 (0.69)	8.60 (0.44)	
**Central American**				
Normal (BMI <25)	28.45 (2.48)	33.42 (2.66)	30.96 (1.75)	26.5 (<.001)
Overweight (BMI 25-29.9)	50.60 (3.44)	28.38 (2.62)	39.39 (2.11)	
Obese (BMI ≥30)	17.77 (2.57)	26.54 (2.25)	22.20 (1.72)	
Data missing	3.18 (1.06)	11.66 (1.74)	7.46 (0.99)	

a SEs (standard errors) adjusted for design effect with SUDAAN.

**Table 3:** Prevalence of Normal Weight, Overweight, and Obesity Among Hispanic Adults, by Age and Ethnic Subgroup, 2001 California Health Interview Survey

Body Mass Index (BMI) Classification	Age 18-29% (SE^a^)	Age 30-39% (SE^a^)	Age 40-49% (SE^a^)	Age 50-64% (SE^a^)	Age ≥65% (SE^a^)	χ^2^_12 _(*P*)
**Mexican American**						
Normal (BMI <25)	43.66 (1.44)	25.86 (1.27)	21.36 (1.25)	17.43 (1.31)	27.65 (2.39)	341.6 (<.001)
Overweight (BMI 25-30)	31.56 (1.29)	39.35 (1.31)	41.58 (1.39)	38.97 (1.59)	36.40 (2.90)	
Obesity (BMI ≥30)	15.68 (0.95)	25.71 (1.31)	31.30 (1.50)	34.19 (1.79)	25.75 (2.91)	
Data missing	9.10 (0.79)	9.09 (0.84)	5.76 (0.72)	9.40 (1.10)	10.20 (1.86)	
**Central American**						
Normal (BMI <25)	42.18 (3.60)	29.54 (3.09)	20.67 (2.67)	28.88 (5.36)	19.54 (6.57)	42.3 (<.001)
Overweight (BMI 25-30)	36.30 (3.98)	45.18 (3.88)	40.64 (3.05)	31.93 (4.98)	39.63 (9.16)	
Obesity (BMI ≥30)	13.97 (3.00)	20.33 (3.39)	32.54 (3.56)	29.62 (4.83)	18.10 (4.90)	
Data missing	7.55 (1.73)	4.96 (1.57)	6.15 (1.76)	9.57 (3.14)	22.74 (9.04)	

a SEs (standard errors) adjusted for design effect with SUDAAN.

**Table 4:** Results of a Multivariate Analysis of Risk for Overweight and Obesity Among Mexican Americans, by Sex and Selected Characteristics, 2001 California Health Interview Survey

Characteristics	Overweight OR (95% CI^a^)		Obesity OR (95% CI^a^)	

	Men	Women	Men	Women
**Age, y**				
18-29	Ref	Ref	Ref	Ref
30-39	2.12 (1.54-2.92)	1.49 (1.10-2.03)	1.97 (1.44-2.70)	1.72 (1.27, 2.32)
40-49	2.35 (1.58-3.48)	1.63 (1.19-2.23)	2.58 (1.88-3.53)	2.12 (1.50-2.99)
50-64	2.52 (1.57-4.05)	2.00 (1.35-2.98)	3.69 (2.56-5.31)	3.00 (2.02-4.43)
≥65	1.15 (0.63-2.10)	1.61 (0.92-2.83)	1.56 (0.94-2.58)	1.22 (0.73-2.06)
**Employed**				
Yes	Ref	Ref	Ref	Ref
No	0.83 (0.57-1.19)	0.94 (0.75-1.17)	1.01 (0.77-1.32)	1.00 (0.77-1.30)
**Education**				
College graduate	Ref	Ref	Ref	Ref
High school graduate or some college	1.21 (0.80-1.84)	1.44 (1.02-2.04)	1.38 (0.87-2.19)	1.29 (0.81-2.05)
<High school graduate	1.02 (0.65-1.61)	1.61 (1.09-2.39)	2.34 (1.39-3.94)	1.96 (1.14-3.37)
**Marital status**				
Married	Ref	Ref	Ref	Ref
Divorced/widowed/separated	1.19 (0.89-1.60)	0.87 (0.65-1.14)	0.85 (0.65-1.11)	0.85 (0.65-1.12)
Single	0.71 (0.52-0.97)	0.88 (0.61-1.27)	0.68 (0.46-0.99)	0.78 (0.49-1.23)
**No. of children**				
0		Ref		Ref
1		1.05 (0.67-1.65)		1.01 (0.64-1.61)
2		1.36 (0.87-2.12)		1.35 (0.82-2.20)
3		1.81 (1.18-2.78)		1.36 (0.84-2.20)
≥4		1.78 (1.11-2.85)		1.91 (1.20-3.04)
**Duration of U.S. residence, y**				
<15	Ref	Ref	Ref	Ref
≥15 or U.S. born	1.28 (0.96-1.70)	1.18 (0.87-1.60)	1.36 (1.01-1.87)	1.35 (0.98-1.86)
**Has health insurance**				
Yes	Ref	Ref	Ref	Ref
No	0.80 (0.60-1.06)	0.88 (0.68-1.15)	0.75 (0.57-0.97)	0.76 (0.59-0.99)
**Self-reported health status (continuous variable)**	1.08 (0.96-1.22)	1.18 (1.05-1.34)	1.66 (1.44-1.91)	1.64 (1.43-1.89)
**Binge drinking**				
No	Ref	Ref	Ref	Ref
Yes	0.95 (0.71-1.26)	1.38 (0.86-2.22)	0.95 (0.56-1.61)	0.95 (0.55-1.65)
**Current smoker**				
No	Ref	Ref	Ref	Ref
Yes	1.11 (0.80-1.55)	1.03 (0.72-1.47)	0.76 (0.51-1.15)	0.75 (0.50-1.13)
**Consumes ≥5 servings of fruits/vegetables per day**				
No	1.24 (0.97-1.59)	1.11 (0.88-1.42)	1.15 (0.88-1.49)	1.18 (0.91-1.53)
Yes	Ref	Ref	Ref	Ref
**Uses vitamin supplements**				
No	1.09 (0.85-1.39)	1.24 (0.98-1.56)	1.31 (1.07-1.62)	1.28 (1.04-1.58)
Yes	Ref	Ref	Ref	Ref
**Engaged in vigorous activity for at least 10 minutes during preceding 30 days**				
No	0.82 (0.62-1.09)	1.51 (1.15-1.98)	1.15 (0.85-1.55)	1.14 (0.84-1.55)
Yes	Ref	Ref	Ref	Ref

OR indicates odds ratio; CI, confidence interval; ref, referent group.

a 95% confidence interval computed on the basis of weighting provided in the California Health Interview Survey.

**Table 5:** Results of a Multivariate Analysis of Risk for Overweight and Obesity Among Central Americans, by Sex and Selected Characteristics, 2001 California Health Interview Survey

Characteristics	Overweight OR (95% CI^a^)		Obesity OR (95% CI^a^)	

	Men	Women	Men	Women
**Age, y**				
18-29	Ref	Ref	Ref	Ref
30-39	2.71 (1.25–5.88)	0.59 (0.29–1.20)	0.88 (0.34–2.30)	2.14 (0.98– 4.69)
40-49	1.68 (0.62–4.57)	2.00 (0.94–4.25)	1.25 (0.37–4.20)	7.76 (3.57–16.87)
50-64	1.20 (0.31–4.57)	1.37 (0.49–3.85)	0.86 (0.17–4.45)	3.06 (1.08–8.64)
≥65	10.41 (2.16–50.14)	2.18 (0.48–9.83)	2.71 (0.30–24.80)	2.60 (0.46–14.87)
**Employed**				
Yes	Ref	Ref	Ref	Ref
No	1.12 (0.44–2.85)	0.72 (0.38–1.37)	0.50 (0.10–2.57)	1.68 (0.90–3.14)
**Education**				
College graduate	Ref	Ref	Ref	Ref
High school graduate or some college	0.41 (0.14–1.27)	1.32 (0.55–3.16)	2.57 (0.61–10.87)	0.30 (0.11–0.80)
<High school graduate	0.32 (0.10–0.99)	2.14 (0.91–5.04)	1.41 (0.27–7.27)	0.95 (0.35–2.57)
**Marital status**				
Married	Ref	Ref	Ref	Ref
Divorced/widowed/separated	0.95 (0.34–2.62)	0.60 (0.30–1.22)	0.75 (0.23–2.51)	0.91 (0.44–1.89)
Single	0.59 (0.27–1.29)	0.60 (0.24–1.50)	0.17 (0.05–0.58)	1.38 (0.65–2.94)
**Number of children**				
0		Ref		Ref
1		0.94 (0.36–2.45)		0.37 (0.12–1.14)
2		0.95 (0.41–2.23)		0.32 (0.11–0.94)
3		2.80 (1.11–7.04)		0.97 (0.32–2.92)
≥4		1.64 (0.58–4.63)		0.71 (0.21–2.39)
**Duration of U.S. residence**				
<15 years	Ref	Ref	Ref	Ref
≥15 years/U.S. born	1.46 (0.76–2.82)	0.72 (0.38–1.39)	4.92 (1.64–14.74)	0.57 (0.27–1.16)
**Has health insurance**				
Yes	Ref	Ref	Ref	Ref
No	2.21 (1.15–4.24)	0.73 (0.39–1.36)	3.80 (1.45–9.95)	0.58 (0.34–1.01)
**Self-reported health status (continuous variable)**	0.78 (0.59–1.03)	0.98 (0.74–1.30)	0.84 (0.54–1.31)	1.36 (0.97–1.91)
**Binge drinking**				
No	Ref	Ref	Ref	Ref
Yes	0.62 (0.36–1.08)	0.59 (0.19–1.86)	0.43 (0.18–0.99)	0.30 (0.07–1.20)
**Current smoker**				
No	Ref	Ref	Ref	Ref
Yes	0.96 (0.42–2.17)	0.94 (0.20–4.47)	1.38 (0.53–3.61)	0.79 (0.23–2.66)
**Consumes ≥5 servings of fruits or vegetables per day**				
No	1.09 (0.60–1.95)	0.97 (0.57–1.64)	1.25 (0.53–2.96)	1.72 (0.91–3.24)
Yes	Ref	Ref	Ref	Ref
**Takes vitamin supplements**				
No	1.75 (0.91–3.36)	1.14 (0.65–1.99)	2.05 (0.90–4.68)	1.72 (0.91–3.24)
Yes	Ref	Ref	Ref	Ref
**Engaged in vigorous activity for at least 10 minutes during preceding 30 days**				
No	0.66 (0.33–1.34)	1.54 (0.72–3.30)	0.98 (0.41–2.39)	0.96 (0.41–2.23)
Yes	Ref	Ref	Ref	Ref

OR indicates odds ratio; CI, confidence interval; ref, referent group.

a 95% confidence interval computed on the basis of weighting provided in the California Health Interview Survey.
